# Phenotypic Heterogeneity in Sugar Utilization by *E. coli* Is Generated by Stochastic Dispersal of the General PTS Protein EI from Polar Clusters

**DOI:** 10.3389/fmicb.2017.02695

**Published:** 2018-01-17

**Authors:** Sutharsan Govindarajan, Nitsan Albocher, Tamar Szoke, Anat Nussbaum-Shochat, Orna Amster-Choder

**Affiliations:** Department of Microbiology and Molecular Genetics, IMRIC, Faculty of Medicine, The Hebrew University of Jerusalem, Jerusalem, Israel

**Keywords:** bacterial polarity, cell poles, proteins localization, dynamic localization, PTS system, general PTS proteins, phenotypic heterogeneity

## Abstract

Although the list of proteins that localize to the bacterial cell poles is constantly growing, little is known about their temporal behavior. EI, a major protein of the phosphotransferase system (PTS) that regulates sugar uptake and metabolism in bacteria, was shown to form clusters at the *Escherichia coli* cell poles. We monitored the localization of EI clusters, as well as diffuse molecules, in space and time during the lifetime of *E. coli* cells. We show that EI distribution and cluster dynamics varies among cells in a population, and that the cluster speed inversely correlates with cluster size. In growing cells, EI is not assembled into clusters in almost 40% of the cells, and the clusters in most remaining cells dynamically relocate within the pole region or between the poles. In non-growing cells, the fraction of cells that contain EI clusters is significantly higher, and dispersal of these clusters is often observed shortly after exiting quiescence. Later, during growth, EI clusters stochastically re-form by assembly of pre-existing dispersed molecules at random time points. Using a fluorescent glucose analog, we found that EI function inversely correlates with clustering and with cluster size. Thus, activity is exerted by dispersed EI molecules, whereas the polar clusters serve as a reservoir of molecules ready to act when needed. Taken together our findings highlight the spatiotemporal distribution of EI as a novel layer of regulation that contributes to the population phenotypic heterogeneity with regard to sugar metabolism, seemingly conferring a survival benefit.

## Introduction

The poles of rod-shaped bacterial cells play an important role in various molecular processes, including DNA segregation, metabolic regulation and aggregate clearance (Bowman et al., 2011; Govindarajan et al., 2012; Laloux and Jacobs-Wagner, 2014). The poles are also emerging as hubs for localization of many proteins, including sensory systems (Alley et al., 1992; Janakiraman and Goldberg, 2004; Briegel et al., 2009; Rudner and Losick, 2010; Amster-Choder, 2011). Currently, a handful of mechanisms have been suggested to underlie targeting of proteins to the poles. These include interaction with the polar anionic lipid cardiolipin, recognition of membrane domains with strong negative curvature, present in the poles and septa, and self-assembly in nucleoid-free spaces (reviewed in Govindarajan et al., 2012; Laloux and Jacobs-Wagner, 2014; Treuner-Lange and Sogaard-Andersen, 2014).

Notably, while the list of proteins that localize to the poles is constantly growing, less is known about their dynamics within or between the poles. Several polar complexes were shown to exhibit dynamic behavior, relocating from pole to pole, from pole to mid-cell, or from lateral sites to the poles, with their spatiotemporal dynamics often linked to developmental pathways (Jensen et al., 2002). A classic example for proteins that exhibit pole-to-pole dynamics is the *Escherichia coli* MinCDE complex, which negatively regulates FtsZ polymerization at the poles and restricts Z-ring formation to mid-cell (Lutkenhaus, 2007). In *Caulobacter crescentus*, the polarity-establishing proteins PopZ and TipN were shown to relocate from one pole to the other in a cell cycle-dependent manner, such that they mark the old and new pole, respectively (Huitema et al., 2006; Lam et al., 2006; Bowman et al., 2008; Ebersbach et al., 2008). FtsZ, which assembles into a ring in mid-cell during division, was recently shown to relocate to the cell poles of non-dividing cells and re-assemble there (Yu et al., 2017). Dynamic localization from lateral sites to the poles was observed for chemoreceptor complexes in *E. coli* (Thiem and Sourjik, 2008).

The bacterial phosphotransferase (PTS) system controls hierarchical uptake and utilization of preferred carbohydrates from complex environments (Deutscher et al., 2006). Additionally, the PTS controls other pathways, including carbon catabolite repression and inducer exclusion (Deutscher et al., 2006). The spatial organization of the PTS system has been studied in our lab. We found that the control center of the PTS system, which comprises the major PTS proteins EI and HPr, localizes to the cell poles of *E. coli*. Although EI and HPr interact with each other, polar localization of each of them occurs independently of the other (Lopian et al., 2010). Moreover, activation of the PTS system was shown to affect the localization of HPr but not of EI. Our recent study, which focused on identifying the mechanism of EI localization revealed that a geometric cue is important for EI targeting (Govindarajan et al., 2013). Thus, similar to DivIVA, a *B. subtilis* negative membrane curvature sensor protein, EI localizes to regions of strong negative curvature in the membrane, which are usually present in the poles and septa (Govindarajan et al., 2013). However, unlike DivIVA, which can directly sense the membrane curvature through its membrane-binding α-helical domain (Lenarcic et al., 2009; Ramamurthi and Losick, 2009; Oliva et al., 2010), the soluble EI protein was suggested to localize to these regions via other, yet unknown, proteins that sense membrane curvature.

In this study, we employed time-lapse fluorescence microscopy in live cells to explore the temporal organization of EI in growing and quiescent *E. coli* cells. We show that polar EI clusters are often dynamic and that their dynamic range differs among cells in the population, with their speed negatively correlating with cluster size. EI dynamics is energy-dependent, since it is negatively affected by inhibition of cell metabolism. EI cluster dynamics does not depend on the type of sugar, whether it is PTS or non-PTS. However, regardless of the sugar source, EI clustering inversely correlates with its function, that is, EI has a higher capacity to be active in cells in which it is uniformly distributed, and its higher-order assembly into clusters prevents its activity. In line with this, during transition from inactive to active state of growth, EI molecules disperse out of the cluster in a significant number of cells in a population. Intriguingly, EI cluster formation is an event that is stochastic in time, which generates phenotypic heterogeneity within a population.

## Results

### EI clusters exhibit several dynamic localization patterns that are energy dependent

**Figure 1:**
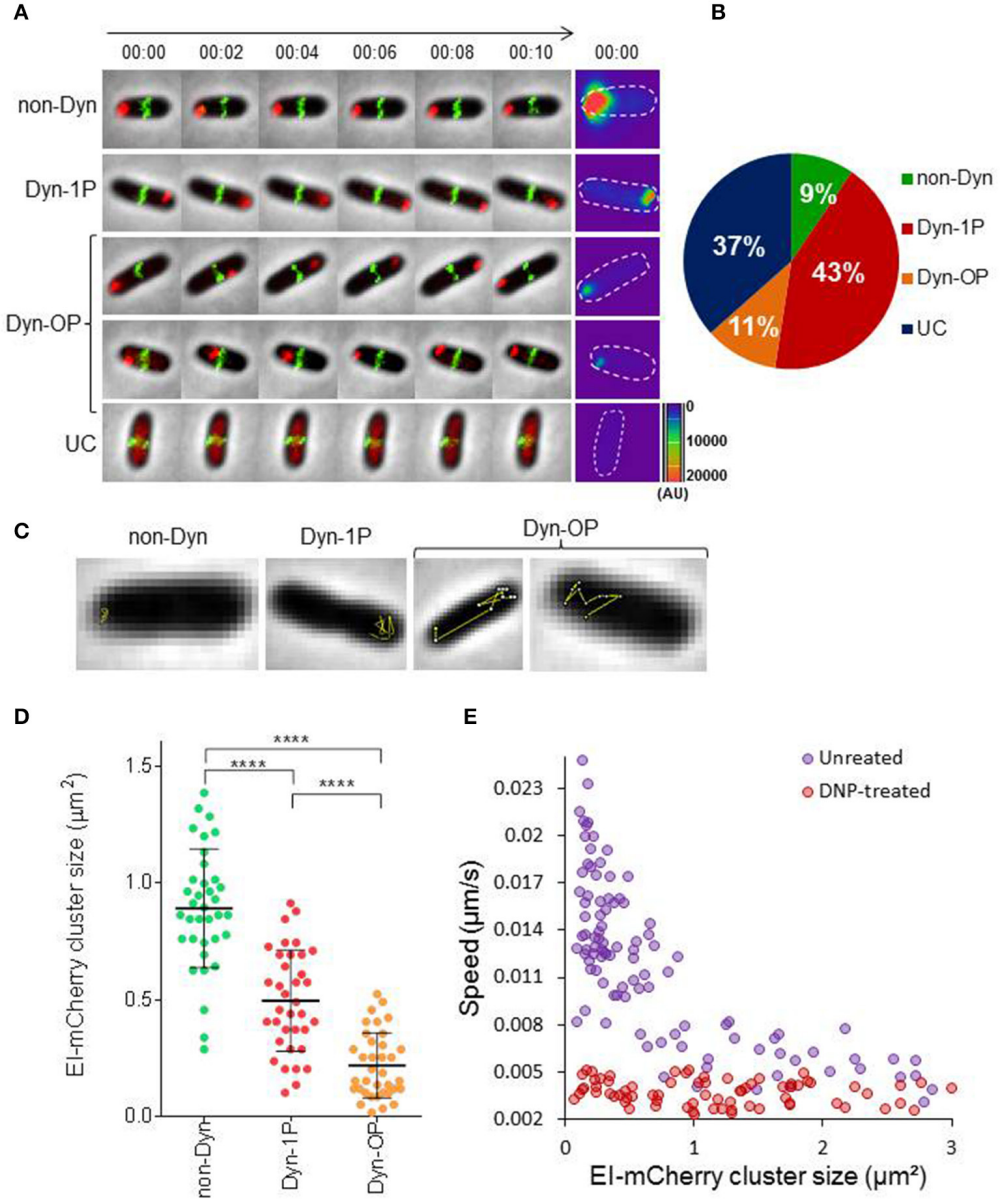
EI is a dynamic protein whose motion is metabolism-dependent **(A)** Time-lapse microscopy images of wild-type *E. coli* cells expressing EI-mCherry and ZapA-GFP. Representative images of the four different patterns of EI dynamics are shown: (a) non-dynamic (non-Dyn), (b) dynamic within one pole (Dyn-1P), (c) dynamic between the two poles or from pole to midcell (Dyn-OP), (d) undetectable cluster (UC). The mCherry and GFP fusion proteins were observed by fluorescence microscopy and cells were observed with phase microscopy. Overlays of the fluorescence signal (GFP, green and mCherry, red) over the phase contrast images (gray) are shown. Surface intensity plots showing the fluorescent intensity (AU) of the EI-mCherry signal at time 0, which corresponds to the cellular distribution of EI-mCherry in each of the cells. The contour of each cell is outlined. **(B)** Pie chart showing the fractions of cells that exhibit the different patterns of EI dynamics, i.e., non-dynamic (non-Dyn, green), dynamic within one pole (Dyn-1P, red), dynamic from pole to midcell or from pole to pole (Dyn-OP, orange), and undetectable cluster (UC, blue), in populations grown to early log in minimal medium supplemented with glucose as carbon source. The Standard division was between 1 and 2%. **(C)** Two-dimensional trajectories of EI-mCherry cluster movement overlaid on the corresponding phase contrast (gray) images. **(D)** Distribution diagram indicating the distribution of EI-mCherry clusters area (μm^2^) in cells (referred to as size) exhibiting the different patterns of EI dynamics (40 cells from each group). *P*-values were obtained by ordinary one-way ANOVA: ^****^p-value < 0.0001. **(E)** Scatter plot of cluster speed (μm/s) vs. EI-mCherry clusters area (μm^2^), drawn for cells treated (red) or not treated (purple) with DNP. See Materials and Methods for experimental details. Spearman correlation for the untreated ρ = −0.78, *p*-value < 10^−8^.

The general PTS protein EI has been shown to form clusters that localize mainly to the poles or to mid-cell (Lopian et al., 2010; Govindarajan et al., 2013). However, the temporal behavior of these clusters, as well as that of EI molecules that are not associated with clusters has not been characterized. We first set out to monitor the spatiotemporal localization of EI in actively growing cells. For this purpose, we monitored exponentially growing cells, which express EI fused to mCherry, as well as ZapA—a marker for septal location (Galli and Gerdes, 2010)—fused to GFP, both expressed from the native chromosomal loci under the control of their respective promoters, by time-lapse microscopy. First, we verified that the activity of the chromosome-encoded EI-mCherry protein is comparable to that of the wild-type protein by comparing the growth rate of the strains expressing EI-mCherry or EI in minimal medium supplemented with PTS sugars (glucose or sorbitol) or with a non-PTS sugar (lactose). The results in Figure S1 show that the growth of the two strains on all sugar sources is alike, indicating that EI-mCherry has a similar activity to EI. Next, cells expressing EI-mCherry and ZapA-GFP were grown in minimal medium supplemented with glucose till they reached OD_600_ = 0.2, and images were acquired every minute for 10 min Based on the presence and the spatiotemporal localization patterns exhibited by EI, we divided the cells to four groups (see Figures 1A,B and Movie S1): (a) Non-dynamic (non-Dyn), that is, the EI cluster was observed in one of the poles without detectable movement (9%); (b) Dynamic within one pole (Dyn-1P), that is, the EI cluster was dynamic within the zone of one pole (43%); (c) Dynamic outside the zone of one pole (Dyn-OP), that is, the EI cluster switched from one pole to another or between the pole and mid-cell (11%); (d) EI clusters were not detected (UC), but, rather, EI molecules appeared as freely diffuse in the cytoplasm (37%) (Figures 1A,B). Two-dimensional trajectories of the movement tracks, presented in Figure 1C, show that, except for cells that exhibited a non-dynamic pattern (non-Dyn), in all cells exhibiting dynamic localization patterns (Dyn-1P and Dyn-OP), EI moved in a zigzag manner, that is, in jagged lines with sharp turns.

Heat map presentation of EI-mCherry fluorescence intensity, made for representative cells at time 0, shows an inverse correlation between the EI cluster intensity and its dynamics (Figure 1A, last panel). Thus, the intensity of non-Dyn clusters is much higher compared to Dyn-1P, and the intensity of the latter is significantly higher than that of Dyn-OP. To quantitatively analyze the relationship between EI dynamics and cluster size at the population level, we plotted the area of EI-mCherry clusters (will be referred to as cluster size) for each localization pattern in the corresponding population (Figure 1D, *n* = 120). The results demonstrate that non-Dyn clusters have the largest area and are non-mobile, Dyn-1P clusters are smaller and they move within a limited zone, and Dyn-OP clusters are the smallest and the most mobile. These results indicate that the dynamics of EI clusters inversely correlates with their size, that is, the bigger the cluster, the more static it is.

Next, we quantitatively analyzed the correlation between the rate of EI clusters movement and their size. To answer this question, we performed time-lapse experiments, during which we acquired images of EI-mCherry localization every 10 s for a total of 5 min. From these images, we calculated the average speed (μm/s) of individual EI-mCherry clusters and plotted it as a function of average EI-mCherry cluster size. The results (Figure 1E, purple circles) show that the average speed of EI cluster inversely correlates with its size (Spearman correlation ρ = −0.78, *p*-value < 10^−8^), that is, clusters with smaller size moved faster than clusters with bigger size.

To figure out whether active cellular metabolism is required for EI motion, we repeated the time-lapse experiment described above with cells treated with Di-nitro phenol (DNP), an inhibitor of cell metabolism (Parry et al., 2014). The results (Figure 1E, red circles) show that movement of EI was restrained in DNP-treated cells, as evidenced by the dramatic reduction in cluster speed, and the lack of correlation between the speed and the cluster size.

Together, the data presented in this section suggest that the motion range and speed of EI clusters inversely correlate with their size and that this motion requires energy.

### EI clusters are formed stochastically in time by assembly of pre-existing dispersed molecules

**Figure 2:**
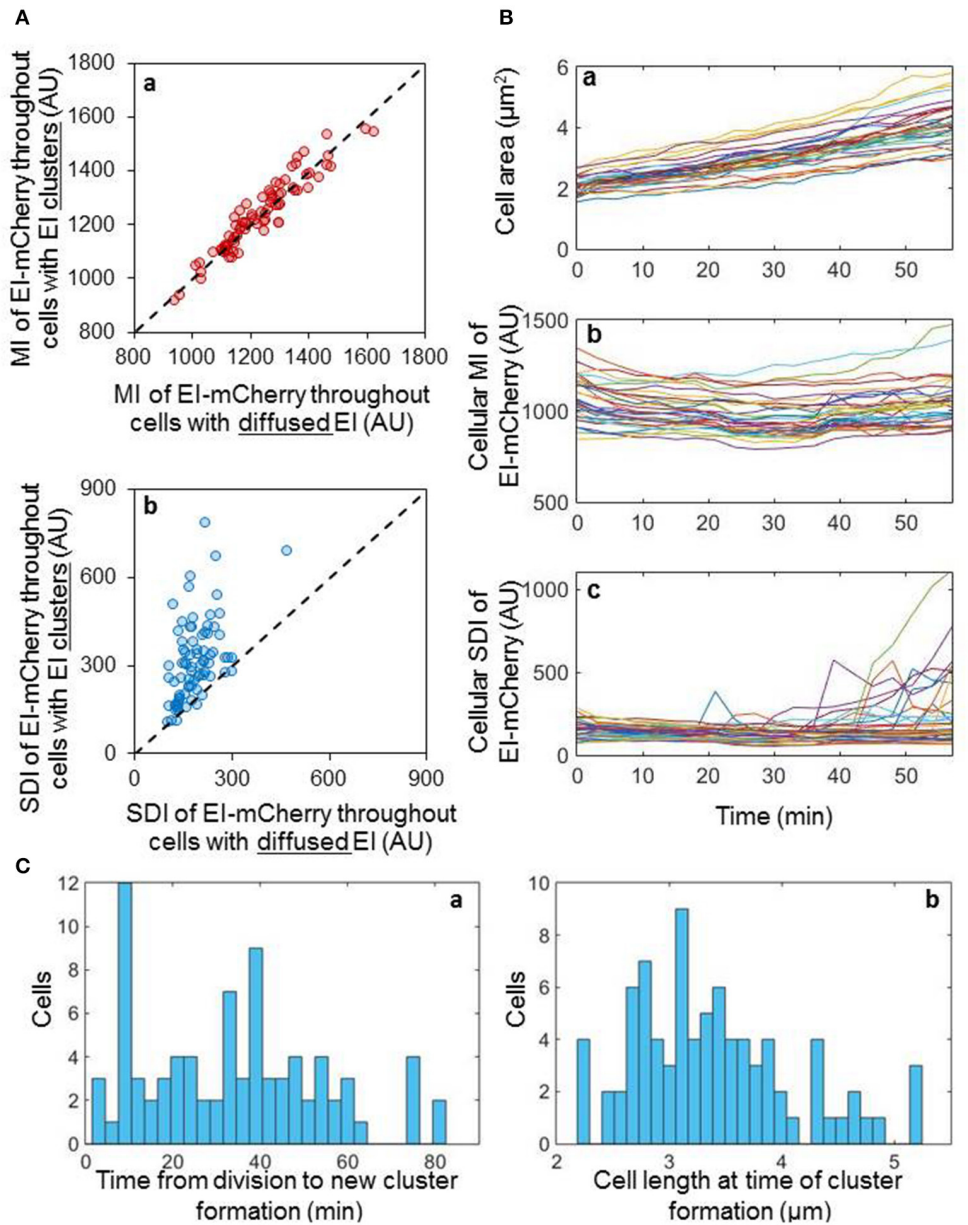
EI clusters are formed by assembly of pre-existing dispersed molecules in a stochastic manner **(A)** Scatter plots of the cellular EI-mCherry mean intensity (MI) **(a)** or the cellular EI-mCherry standard deviation intensity (SDI) **(b)** for individual cells before cluster formation vs. after cluster formation (*n* = 78). Dashed line, *y* = *x*. **(B)** Line charts showing the area **(a)**, MI **(b)** and SDI **(c)** of individual cells, in which EI-mCherry appeared diffuse at time 0, over 60 min (*n* = 36). **(C)** Distribution of the time it takes a new cluster to form from the time each cell has divided **(a)** and distribution of the cell length at the time a new cluster has formed in each cell **(b)**.

To shed light on the process of EI cluster formation, we asked whether EI clusters form by *de novo* protein synthesis or by assembly of pre-existing molecules. We assumed that if new clusters nucleate as a result of expression burst, then the amount of EI before cluster formation would be lower than its amount after cluster formation. On the other hand, if new clusters are formed by assembly of pre-existing molecules, the amount of EI before and after cluster formation are expected to be comparable. To distinguish between these possibilities, we followed, by time-lapse microscopy, the process of EI cluster formation in 78 cells, and quantified the mean intensity (MI) and the standard deviation intensity (SDI) of the EI-mCherry fluorescent signal before and after cluster formation (Figure 2A). Of note, whereas the MI estimates the amount of EI-mCherry in the cell, the SDI provides information on the distribution of EI-mCherry within the cell i.e., whether or not a cluster has been formed. Moreover, Figure S2 shows that the size of EI-mCherry clusters directly correlates with cellular EI mCherry SDI (Pearson correlation ρ = 0.83 *P*-value = 7.89E-34). This implies that the cluster size and the cellular distribution of the EI-mCherry signal directly correlate and, hence, we can use the SDI of the signal as an estimate for cluster size. Thus, cells with low SDI value have diffuse EI molecules, while cells with high SDI value have highly clustered EI. As shown in Figure 2Aa, the mean fluorescence intensity of EI-mCherry of individual cells, before and after cluster formation, are comparable, indicating that the new clusters are assembled from pre-existing molecules. We then plotted the SDI of EI-mCherry before and after cluster formation, one against the other. The results in Figure 2Ab show that the SDI fluorescence intensity values of EI-mCherry after cluster formation were higher than the values before cluster formation in the majority of cells, indicating that new clusters are indeed formed in these cells.

Together, the results in Figures 2Aa,b imply that the new clusters are formed from pre-existing molecules. To validate this observation, we tracked the distribution and the concentration of EI-mCherry molecules in 36 individual cells, in which this protein appeared diffuse at time 0, for 60 min. To determine the cell cycle status of the cells, we also measured the area of these cells. The results presented in Figure 2Ba show that the growth rate was similar in all the tracked cells. Moreover, the EI-mCherry MI did not change much during cell growth, i.e., was similar in all time points (Figure 2Bb). However, in 14 cells, the EI-mCherry SDI abruptly increased at different time points, indicating that new clusters are formed in these cells during the time course of the experiment (Figure 2Bc).

The results in Figures 2A,B suggest that the freely diffuse EI molecules assemble rapidly and haphazardly to form higher order clusters within the cytoplasm in a manner that is independent of EI-mCherry level, which does not fluctuate. Of note, the results in Figure 2B were not obtained with synchronized cells. To test whether there is any linkage between cluster formation and time or cell cycle, we followed newly divided cells (*n* = 80), in which EI-mCherry molecules appeared diffuse, and monitored the time at which the clusters formed (Figure 2Ca). Additionally, the length of the cell, which provides information on the cell cycle status, at the time of cluster formation was also measured (Figure 2Cb). As shown in Figures 2Ca,b, the time of cluster formation and the length of the cell during cluster formation were very different in these cells, which were synchronized according to their cell cycle, indicating that new EI clusters are formed stochastically during the cell cycle. Of note, the stochasticity is deduced from the fact that we did not find any cellular parameter that is linked to EI clustering. The possibility that EI clusters are formed in response to some unknown intracellular change exist, but this putative change, in turn, is seemingly not linked to cell growth and cell cycle and shows stochasticity in time. Taken together, our results show that *E. coli* populations are heterogonous with respect to whether and when EI clusters are formed during growth.

### Diffuse EI molecules dynamically incorporate into polar clusters

**Figure 3:**
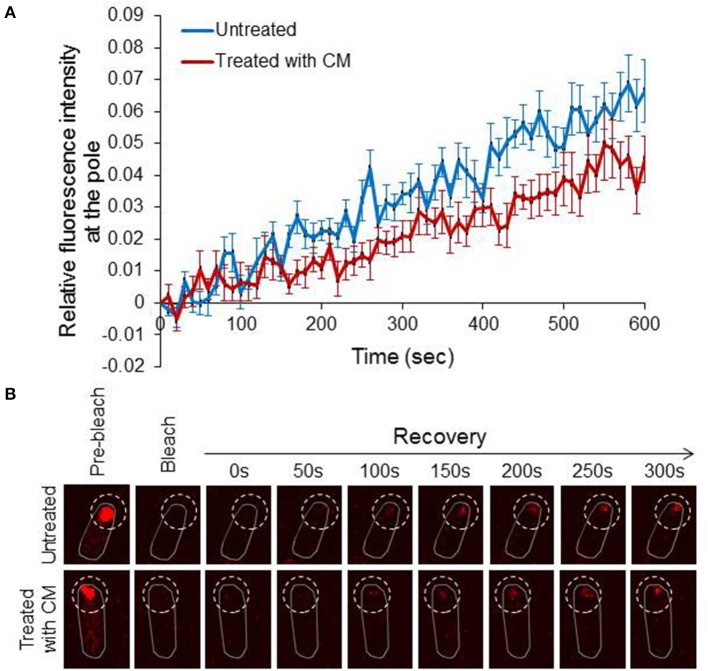
Diffuse EI molecules dynamically incorporate into polar clusters **(A)** Line chart showing the recovery of EI-mCherry fluorescence signal in untreated cells (blue) (*n* = 14) or in chloramphenicol-treated cells (red) (*n* = 12) from the time of bleaching (time 0) over 600 s. Mean and standard error are shown. **(B)** Representative FRAP microscopy images of wild-type *E. coli* cells expressing EI-mCherry in the absence of treatment (upper panel) or in the presence of chloramphenicol (lower panel). Images at selective time points are shown. The contour of the bleached area in the fluorescent images is outlined.

After showing that diffuse EI molecules can assemble to form a new cluster, we asked whether these molecules can join a pre-formed EI cluster. To answer this question we performed Fluorescence Recovery After Photobleaching (FRAP). For this purpose, we photobleached the entire pole area, where the clusters are localized, and the recovery of fluorescence intensity at the pole was measured as a function of time and plotted relative to the time after bleaching. For the analysis, we chose cells with big EI clusters that were non-dynamic before photobleaching. Furthermore, in order to distinguish between recovery by assembly of pre-existing diffuse molecules and recovery by assembly of newly synthesized molecules, we performed another set of FRAP with cells spotted on an agar pad that contained the translation inhibitor chloramphenicol (CM). The results in Figure 3A show that nearly 6% of EI-mCherry fluorescent signal in the pole area was recovered after 600 s in untreated cells (*n* = 14), whereas the recovery of the fluorescent signal in the CM-treated cells during this time was slightly lower i.e., nearly 4% (*n* = 12). That is, about one third of the recovered EI-mCherry at the pole were *de novo* synthesized. Of note, the time it took the polar signal to appear and the pace it developed in the untreated and CM-treated samples were very similar (Figure 3B). These results indicate that the majority of molecules that assemble with the clusters are pre-existing diffuse EI-mCherry molecules, which were present outside the bleached area.

### Growth phase, but not the carbon source in the growth medium, affects formation of EI clusters

**Figure 4:**
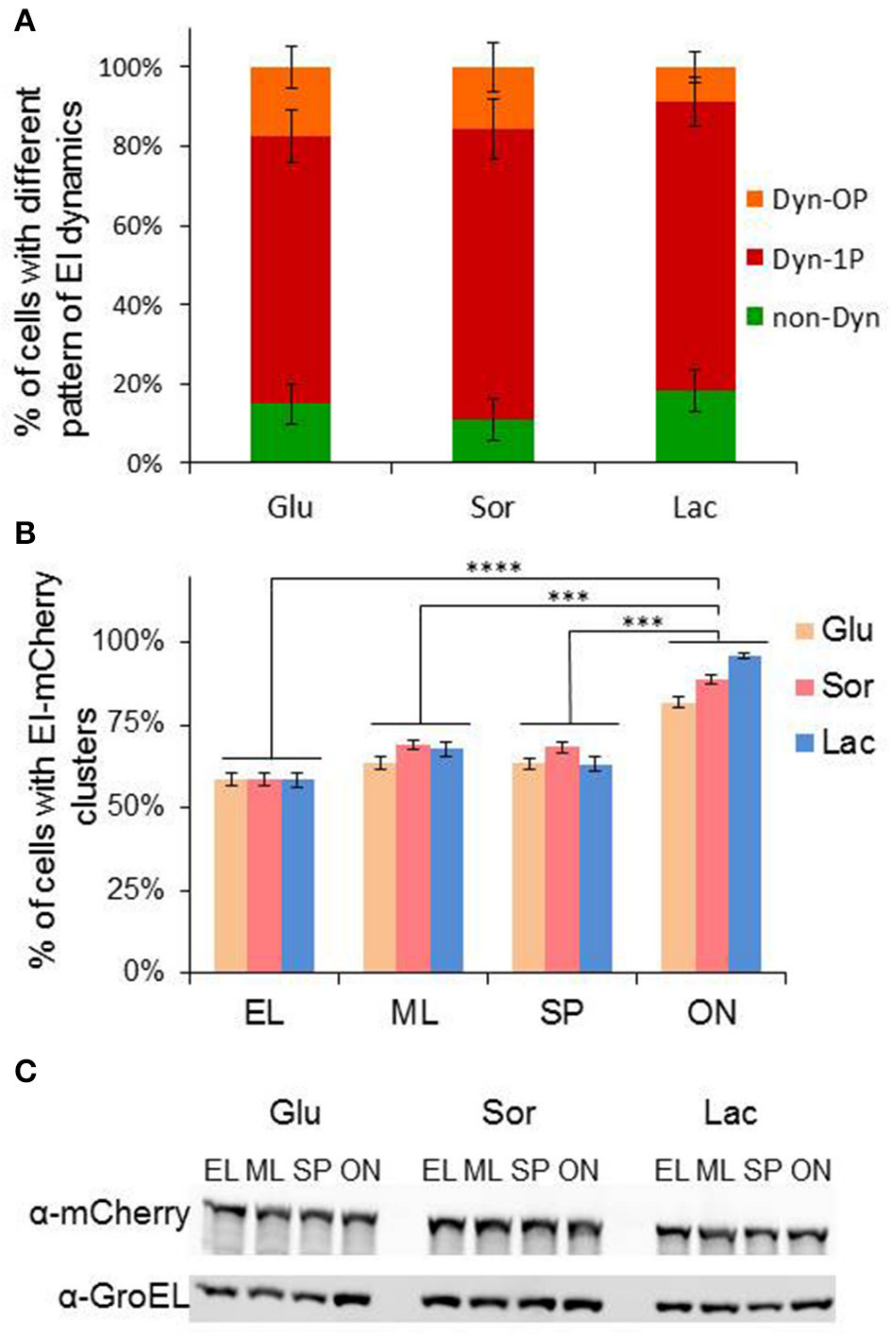
Growth phase, but not the carbon source in the growth medium, affects formation of EI clusters**. (A)** Stacked column chart showing the fractions of cells that exhibit a certain pattern of EI dynamics, i.e., non-dynamic (non-Dyn, green), dynamic within one pole (Dyn-1P, red) and dynamic from pole to midcell or from pole to pole (Dyn-OP, orange), in populations grown to early logarithmic phase in minimal medium supplemented with glucose (Glu, *n* = 328), sorbitol (Sor, *n* = 311) or lactose (Lac, *n* = 286). Cells from three independent experiments were analyzed. Ordinary one-way ANOVA indicated no significant variations among the samples. **(B)** Column chart of the fraction of cells with detectable clusters in cultures grown on different carbon sources glucose (Glu), sorbitol (Sor) or lactose (Lac) at different growth phases: early log (EL; OD_600_ 0.2–0.25), mid-log (ML; OD_600_ 0.5–0.55), early stationary phase (SP; OD_600_ 1.00–1.1) and after overnight growth (ON). More than 400 cells were analyzed from three independent experiments. *P*-values were obtained by ordinary one-way ANOVA: ^***^p-value < 0.001; ^****^p-value < 0.0001. **(C)** Western blot analysis showing the level of EI-mCherry and GroEL proteins, detected by α-mCherry and α-GroEL antibodies, respectively. Cells were grown on different carbon sources to different growth phases, as in **(B)**. Equal amount of samples were collected at specific growth phases, separated on 10% SDS polyacrylamide gel, blotted onto nitrocellulose membrane and detected by the two antibodies.

To determine whether dynamic localization of EI depends on the presence of PTS sugars, we followed EI cluster dynamics in minimal medium supplemented with either glucose or sorbitol, which are PTS sugars, or with lactose, which is a non-PTS sugar, as a sole carbon source, and quantified the fraction of cells in each of the groups that exhibit the different patterns of EI dynamic localization. The results in Figure 4A show that EI clusters were similarly distributed between the groups when cells were grown in glucose or sorbitol containing media, and were only slightly and not significantly different in cells grown in lactose containing media. Hence, the spatiotemporal behavior of EI does not depend on the type of sugar in the growth medium and does not really change between cells grown in the presence of PTS and non-PTS sugars.

Next, we asked whether formation of EI cluster depends on the growth state of the cells. To this end, we grew EI-mCherry-expressing cells in non-PTS medium (minimal medium with lactose) or in PTS medium (minimal medium with glucose or sorbitol) and calculated the percentage of cells with EI clusters at early logarithmic (EL), mid logarithmic (ML), early stationary phase (SP), and late stationary phase (after overnight growth, ON). As shown in Figure 4B, in all three growth media, nearly 60% of cells in EL growth phase formed EI clusters, and this percentage increased a bit upon transition from EL to ML (65–70%) and remained similar during early SP. Conversely, the percentage of cells with EI clusters increased significantly in ON cultures (80–95%), with cells grown in lactose medium containing the highest percentage of clusters. The difference in the percentage of cells with EI clusters in ON cells is not due to an increase in the cellular amount of EI, since Western blot analysis, using α-mCherry antibodies, show that the amount of EI-mCherry present in cells at the different growth phases and in the different growth media, is largely comparable, similar to the housekeeping control protein GroEL (Figure 4C). Therefore, the percentage of cells with EI clusters in the population is growth phase-dependent and is the highest in quiescent cells.

### The process of EI cluster dispersal occurs after exiting quiescence

**Figure 5:**
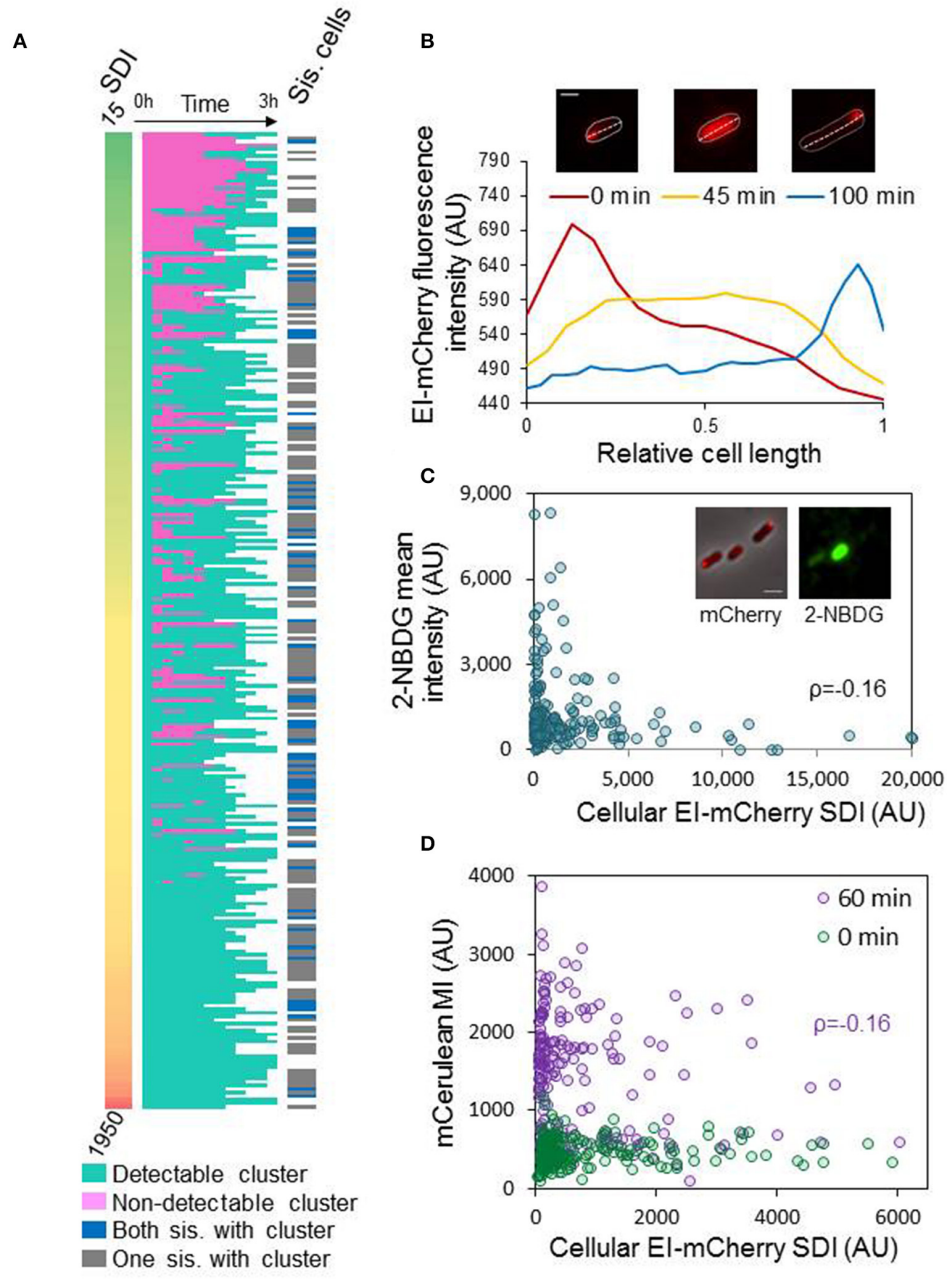
Assembly of EI into clusters affects EI function and dynamics **(A)** Diluted ON cultures were spotted on an agar pad with fresh medium and the cells were observed at time 0, and every 15 min till they divided or for 3 h, by time-lapse microscopy. Left panels: heat map of standard deviation intensity (SDI) of EI-mCherry at time 0. Middle panels: heat map of cells with (turquoise) or without (pink) detectable EI-mCherry clusters over time, from time 0 till cell division (white, time after cell division). Right panels: Heat map of dividing cells with a cluster in each sister cell (blue) or only in one of the sister cells (gray) after 3 h; white rows denote cells that did not divide within the 3 h. **(B)** The images show a representative cell from the ON population, which has an EI-mCherry cluster at time 0 that completely disperses during growth (see image after 45 min) and later reforms (see image after 100 min). The contour of the cell is outlined. Scale bar corresponds to 1 μm. The plot shows the EI-mCherry intensity in this cell vs. the relative cell length at the different time points at which the images were taken (0, 45, and 100 min). **(C)** The images show a representative cells from the ON population, which express EI-mCherry, that were incubated with the glucose analog 2-NBDG. The mCherry fusion protein (red) and the 2-NBDG (green) were observed by fluorescence microscopy and are shown over the phase contrast images (gray) that were observed with phase microscopy. Scale bar 2 μm. The scatter plot presents the 2-NBDG mean intensity (AU) vs. the EI-mCherry SDI (AU) (*n* = 194). Pearson correlation ρ = 0.16; *p*-value = 0.02. **(D)** The scatter plot shows the mean intensity (MI) of mCerulean expressed from Pnag, vs. the cellular EI-mCherry SDI (AU) at the moment of transition to NAG-continuing medium (0 min; green cycles; *n* = 222) and after an hour (60 min; purple cycles; *n* = 274). Pearson correlation for the “60 min” plot ρ = −0.16; *p*-value = 0.007. Pearson correlation for the “0 min” plot ρ = 0.06; *p*-value = 0.366.

The finding that more ON cells contain EI clusters than growing cells (Figure 4B) raised the question of what happens during the transition from stationary to logarithmic phase. Do clusters disperse or a fraction of new cells are born without clusters? To address this question, we spotted diluted ON culture on an agar pad with fresh medium and observed the cells for 3 h by time-lapse microscopy. At the time that the cells were spotted on the agar pad (time 0), we monitored the distribution (SDI) of the EI-mCherry fluorescent signal in 269 cells (Figure 5A, left panel). Of note, as explained above, cluster size can be inferred from the SDI (Figure S2). From this time point and on, we classified the cells to cells having or not having detectable clusters at each time point till they divided (Figure 5A, middle panels). Together, the results in the left and middle panels of Figure 5A show that the population contained the following sub-populations: (i) A relatively small sub-population with no detectable clusters at time 0 (13%), which formed clusters during cell growth in the vast majority of cases. (ii) A very big sub-population of cells with relatively small clusters at time 0 (64%) that dispersed during growth and reformed (35%, see such a cell in Figure 5B) or did not disperse (29%) till they divided. (iii) A medium size sub-population of cells with big clusters at time 0 (23%) that remained throughout the experiment.

To negate the possibility of other factors, such as mechanical perturbations upon shifting cells from liquid media to solid media, contribute to cluster disassembly, we performed a control experiment in which ON cells were spotted on an agar pad made from overnight filtered medium (*n* = 255). In this setup, none of the EI clusters dispersed and only 4% of the cells divided during the 3 h time-lapse microscopy, suggesting that dispersal of EI clusters is specific to transition from quiescence to active growth (Figure S3).

To better understand the nature of the transition from ON to EL phase regarding EI distribution, we followed EI cluster size by tracking the SDI of 10 cells from each of the three sub-populations in Figure 5A. The results of this analysis are presented in Figure S4. The left panel shows the SDI at time 0, using the same scale for all sub-populations. The middle panel shows whether and when the cells had a detectable cluster at each time point till they divided. The right panel shows the SDI at each time point till division, using a different scale for each sub population. By and large, the results show a common trend for each of the first two groups. The SDI of cells in sub-population (i), which had no detectable clusters when they exited quiescence (upper panels; purple shades), remains low till cluster formation, but, upon cluster formation, the SDI doubles, suggesting that cluster formation is a rapid event, although cluster size continues to increase. In cells from sub-population (ii), which had relatively small clusters when exiting quiescence (middle panels; blue shades), there is a moderate decrease in SDI values, even in cells with clusters that remained detectable throughout the experiment, suggesting that cluster dispersal is a continuous process, rather than an abrupt event. With time, the SDI value start to increases. The SDI in cells from sub-population (iii), which had big clusters after exiting quiescence (lower panels; green shades), showed fluctuations with no consistent trend, maybe because big clusters are old and do not disperse completely, or because there is more noise in our measurements in this range. Together, these results suggest that, unlike the abrupt event of cluster formation that occurs at random time points during the cell cycle, dispersal of EI from the cluster is a gradual process, which most cells are going through when exiting quiescence, although it might not be completed, since in some cells EI molecules start to join the cluster before its complete dispersal. Therefore, the process of cluster assembly seems stochastic in time, whereas disassembly is probably due to active sensing, together generating a heterogeneous population.

Because all cells except for two had detectable EI-mCherry clusters during their lifetime, we tested the possibility that cluster formation promote division by measuring the time of cluster formation relative to the cell cycle duration. The results in Figure S5A show no linkage between cluster formation and the cell cycle stage (*n* = 138). Moreover, we did not observe a significant difference in cell cycle duration between cells which were born with or without a cluster (Figure S5B). Finally, 15% of the cells which were born without cluster divide without forming a cluster. Therefore, cluster formation does not seem to promote division. The reason that 2 cells out of 269 cells recovering from ON did not form a cluster (Figure 5A) is probably due to long cell cycle duration. Still, the possibility that EI cluster formation is linked to cell division can be revisited if EI mutants that do not form clusters become available.

Since most cells divided within the timespan of the experiment (3 h), we also addressed the question of what is the fraction of cells that are born without clusters. Hence, we asked if both sister cells had clusters after cell division or only one. The results in Figure 5A show that all cells but two had clusters before division, and that 72% of the sister-cell pairs had only one cell with a cluster (Figure 5A, right panel, gray) and in 28% of the cases both sister cells had a cluster (Figure 5A, right panel, blue) at the end of the time-lapse experiment, implying that 64% of the cells after doubling of the population had clusters. Taking into consideration that at the beginning of the experiment 87% of the cells had clusters, the percentage of clusters in the population dropped after division. Summarily, two phenomena were observed when closely following cells that emerge from stationary phase: the majority of newly added cells lack detectable clusters, suggesting that all EI molecules are dispersed and active at the beginning of growth, and EI clusters in most cells form and disperse at random times during growth, yielding a phenotypically heterogeneous population.

### Clustering of EI inversely correlates with EI function

The process of EI cluster dispersal and the random events of cluster formation, characterized above, are probably general rather than sugar-specific, as there were more EI clusters in ON cultures than in the EL, ML, and SP cultures, independent of the carbon source (Figure 4B). We hypothesized that when the carbon source in the medium is consumed and EI activity is not required, it tends to form clusters that can disperse when a fresh carbon source is supplied and EI needs to function. To explore the possible correlation between EI distribution and function, we incubated overnight cells with 2-NBDG, a fluorescent glucose analog, for 10 min. After washing the cells, images were acquired to quantify the clusters of EI-mCherry and the amount of 2-NBDG in each cell. The scatter plot of EI-mCherry SDI against 2-NBDG mean fluorescence, shown in Figure 5C, suggests that EI has a higher capacity to be active (higher 2-NBDG signal) in cells with lower SDI of EI-mCherry, i.e., cells in which EI-mCherry is distributed uniformly or form small clusters. These results imply that EI is less active when it is clustered in higher-order assemblies and, thus, that its function inversely correlates with clustering and with cluster size (Pearson correlation ρ = −0.16; *p* value = 0.02).

Since 2-NBDG is metabolized to a non-fluorescent product (Yoshioka et al., 1996), we decided to test the correlation between EI distribution and function by another approach. For this purpose, we constructed a strain that contains an EI-mCherry fusion, expressed from the native chromosomal locus, as well as mCerulean gene under the regulation of N-acetyl glucosamine (NAG) promoter (*Pnag*). To induce expression from *Pnag* promoter, phosphorylated NAG needs to be present inside the cell (Yamada and Saier, 1988; Westermayer et al., 2016). Since EI is the first protein in the phosphorylation cascade that enables uptake and phosphorylation of PTS sugars, including NAG (Yamada and Saier, 1988), expression of mCerulean implies that EI is active. The constructed strain, which was initially grown in minimal medium containing glycerol until EL phase, was resuspended in fresh minimal medium containing NAG. Fluorescent images were acquired in order to quantify the expression of EI-mCherry and mCerulean proteins, at time 0 and 60 min. The expression of mCerulean from *Pnag* (mean intensity of Pnag-mCerulean) as well as the distribution of EI-mCherry in the cell (SDI of EI-mCherry) were plotted against each other. As shown in Figure 5D, at time 0 min, at which point cells which were grown only in the presence of glycerol, induction of Pnag-mCerulean could not be observed. In contrast, expression of Pnag-mCerulean was clearly induced when cells were grown in the presence of NAG for 60 min. More importantly, the scatter plots in Figure 5D suggest that EI has a higher capacity to be active (higher mCerulean production) in cells with lower SDI of EI-mCherry i.e., those cells in which EI-mCherry is distributed uniformly or form small clusters. This suggests that EI is relatively less active when it clusters to form higher-order assemblies. Although the possibility of other factors, for example, changes in the uptake or degradation of the sugars, contributing to the observed heterogeneity cannot be completely ruled out, the results obtained with the two assays which tested EI activity in different manners, suggest that EI activity inversely correlates with clustering.

## Discussion

Dynamic organization of proteins in higher-order assemblies, provides a versatile and attractive mechanism for metabolic regulation. In this study, by using time-lapse microscopy as a primary tool, we demonstrate for the first time that the temporal distribution of a central metabolic enzyme, the *E. coli* general PTS protein EI, is multimodal, and that EI clustering inversely correlates with its enzymatic activity. We also show significant repositioning of EI molecules from being in a cluster to being in a diffuse state during transition from inactive to active state of growth, highlighting the importance of EI cluster formation for future need of sugar metabolism.

Our data show that EI clusters, observed as mostly polar in snap-shots in previous studies (Lopian et al., 2010; Govindarajan et al., 2013), exhibit rapid and continuous relocation within the pole region, between the two poles or between the pole and midcell. Thus, the EI clusters spend most of the time in the poles or pole-to-be and a small fraction of their time in relocating among or within these sites, explaining their observation as merely polar in snap-shots. Still, a significant fraction of cells (nearly 11%) contain an EI cluster that remains static at one pole. The demonstration that the mobility of newly formed EI clusters is not restricted to a single pole region, whereas that of bigger clusters is restricted only to the pole region till they become non-dynamic is in line with our finding that the motion and speed of EI clusters is influenced by their size. It has recently been suggested that dynamics of macromolecular complexes within the bacterial cytoplasm inversely correlates with their size (Parry et al., 2014). Our observation of inverse correlation between the size of the EI clusters and their dynamics is in agreement with this.

We have previously shown that EI clusters are located at or near negatively curved regions, although the EI soluble protein, which cannot be anchored in the membrane, localizes to these regions via other, yet unknown, proteins that sense membrane curvature (Govindarajan et al., 2013). Of note, EI is not the first polar protein reported to form at negatively curved membrane regions and to exhibit dynamics. The membrane-anchored chemoreceptors, recently shown to also recognize negative curvature (Strahl et al., 2015), exhibit dynamic cluster positions within the polar zone, which can be more subtle (Studdert and Parkinson, 2005) or less (Thiem et al., 2007). Another protein that senses membrane curvature, but shows dynamics is MreB (Izoré and van den Ent, 2017). Together, these examples highlight the fact that capture of proteins at specific sites, including the poles, is transient, with the degree of transience depending of the nature of the interactions. In light of these findings, dynamics of a soluble protein, such as EI, despite its association with a yet unknown curvature-sensing protein, is less surprising.

EI is the first protein in the PTS system cascade, which controls preferential use of sugars, i.e., preferred use of PTS sugars over non-PTS sugar. Still, EI is important also for uptake of non-PTS sugars, since phosphorylated IIA^glc^, which receives the phosphate from EI via HPr, is required for the activation of adenylate cyclase, whose product is required for the transcription of non-PTS catabolic operons (Deutscher et al., 2014). Indeed, we show that the nature of the sugar, whether it is PTS or non-PTS, does not affect the mobility of EI clusters. Moreover, our results suggest that EI assembly into clusters and disassembly is a mechanism to control its activity in sugar metabolism, independent of the nature of the sugar. This is deduced from the finding that the majority of cells in overnight cultures that grew in the presence of PTS or non-PTS sugars, which apparently exhausted the sugar source and are not actively growing, contained more EI clusters compared to cells in the other growth phases, suggesting that assembly of EI into clusters reflects a non-functional state. We provide a support for this hypothesis by showing that in a significant percentage of cells from overnight cultures (35%), the EI clusters break down and disperse in the cytoplasm shortly after inoculation into a fresh medium. However, at later time points within the cell cycle, new clusters are formed at random time points, in what seem like rapid and non-synchronized events. The negative relationship between EI clustering and sugar uptake is deduced from our demonstration that the intracellular level of a fluorescent glucose analog, as well as the expression of a PTS-dependent promoter inversely correlate with EI clustering. Having said that, disassembly and reassembly of EI clusters were not observed in all cells. It is possible that some cells have an existing pool of disperse active cytoplasmic EI, not detectable in our experiments, whereas EI clusters below our detection capability may exist in other cells, serving as “nucleation centers” for EI higher-order assemblies.

Taken together, our data suggests that EI molecules that are not needed at a given moment join the cluster, from which they can easily disperse when needed. The finding that 63% of the cells in exponential phase have clusters, in addition to diffuse molecules, imply that most cells have more EI than needed under the conditions tested. Since EI is involved in various processes beyond PTS sugar uptake, such as chemotaxis, catabolite repression and utilization of nutrients such as nitrogen and potassium (Lüttmann et al., 2015), our data suggest that the cells anticipate higher need of EI. Hence, the capability of EI clusters to assemble and disassemble contributes to dynamic regulation of the current and expected cellular metabolism.

Many cases of bacterial proteins, whose higher-order assembly hinder their active site or prevent a conformational change required for their activity, have been documented. One example is the CTP synthetase, whose large scale polymerization keeps a subpopulation of the protein in a conformationally restricted form, which can be readily activated (Ingerson-Mahar et al., 2010; Barry et al., 2014). Another example is MurG, an enzyme involved in peptidoglycan subunit synthesis that, when produced in excess, is sequestered to the poles in an inactive form, which, upon need, can be released to the cytoplasm in an active form (Michaelis and Gitai, 2010). More recently, FtsZ was also demonstrated to assemble at the cell poles of non-dividing cells, in the form of a quiescent body, and only upon sensing growth-supporting conditions, the pole-localized FtsZ structures disassemble to form active molecules that engage in cell division (Yu et al., 2017). In all of these cases, the proteins are not degraded when they are not required, but, rather, are kept as a dynamic storage depots, which can be used in the future. Of note, we have previously reported that cluster formation of *B. subtilis* EI occurs only upon growth in a medium supplemented with mannitol as the carbon source and not in a rich medium, such as LB, in which it appeared diffuse (Govindarajan et al., 2013). Hence, it appears that the dynamic clustering property of EI is a general phenomenon, which holds for Gram positive bacteria as well.

Populations of genetically uniform microorganisms, bacterial and yeast cells alike, were shown to exhibit phenotypic heterogeneity with regard to fitness-determining traits (e.g., Gefen and Balaban, 2009; Holland et al., 2014; Wang et al., 2014). This phenomenon, vastly studied with respect to variability among bacterial cells in their resistance to antibiotics, is considered a population-based strategy that can be beneficial to a growing population. The mechanisms that underlie adaptation to stress by heteroresistance are not fully understood at the single cell level. Population heterogeneity was also studied with respect to metabolism, which continuously adapts to unpredictable environmental changes. Upon nutrient change, including that of carbon source, a homogeneous *E. coli* population was shown to split into a growing and a non-growing persisters, which was suggested to stem from stochastic variation in metabolic flux (Kotte et al., 2014). We show that bacterial populations are composed of phenotypic subsets with respect to cluster mobility of a major carbon metabolic enzyme, EI, which can be explained by difference in cluster size. However, the level of heterogeneity is revealed when observing single cells in the population. Thus, despite our general observation that more cells in a quiescent population contain EI clusters than in a growing population, we show that cluster formation is a stochastic event that happen at random timings in individual cells and is not tied up to cell cycle. This stochasticity gains further importance in light of our finding that cluster formation inversely correlates with enzyme activity. Recent studies have shown that biochemical processes are inherently stochastic and cause molecule abundances to fluctuate (Elowitz et al., 2002), a phenomenon that can be used to generate distinct phenotypes (Balaban et al., 2004; Losick and Desplan, 2008). Our findings provide a new organizational explanation to enzymatic heterogeneity, which can hold also for other cellular pathways. Moreover, different reported cases of phenotypic heterogeneity might be linked in nature, as suggested by the observation that uptake of sugars and amino acids increases uptake of various antibiotics (Franklin and Godfrey, 1965; Peng et al., 2015).

## Experimental procedures

### Bacterial strains and growth media

Unless otherwise indicated, overnight cultures were grown in LB supplemented with appropriate antibiotics at 30°C. M9 medium, supplemented with glucose, sorbitol or lactose, were used for time-lapse microscopy experiments. For inhibition of cell metabolism, Dinitrophenol (DNP) at a concentration of 2 mM, was added to the growth medium. When appropriate, antibiotics were added at the following concentrations: kanamycin (30 μg/ml) and chloramphenicol (25 μg/ml) (Sigma-Aldrich). MG1655Φ(*ptsI*-mCherry)strain expressing EI-mCherry from the native chromosomal locus was described previously (Lopian et al., 2010). SUT201, which expresses EI-mCherry and ZapA-GFP from the chromosome, was constructed by P1 transduction of *zapA-gfp(::cat)* from HC261 (Peters et al., 2011) to MG1655Φ(*ptsI*-mCherry). NTS102, which expresses EI-mCherry, *Psrl*-mVenus and *Pnag*-mCerulean from the chromosome, was constructed by P1 transduction of MG1655Φ(*ptsI*-mCherry), linked to KanR, to T1683 (Westermayer et al., 2016).

### Growth conditions used for snap-shot and time-lapse imaging

Ariekacells^R^ coverslip cell chamber (SC15012) was used for time-lapse imaging. Overnight cultures were diluted 1:100 in fresh M9 medium and cells were allowed to grow at 30°C, unless otherwise indicated, till they reached OD_600_ of 0.2. Samples were then spotted on 1% agarose pads, prepared with the respective minimal medium and supplemented with the appropriate sugar which had been pre-equilibrated to 30°C, and imaged immediately by time-lapse microscopy. For calculating the fraction of each dynamic group, time-lapse images were acquired every 3 min for a total of 1 h. For FRAP microscopy, cells were grown in M9 glucose media as described above, and samples were spotted on 1% M9 glucose agarose pads without or with chloramphenicol (25 μg/ml). To measure the correlation of cluster speed vs. EI-mCherry cluster size, cultures grown in fresh M9 glucose medium until OD_600_ of 0.2 were spotted on 1% M9 glucose agar pad and time-lapse images were acquired every 10 s for a total of 5 min. For calculating the fraction of cells with detectable EI clusters, MG1655Φ(*ptsI*-mCherry) cells were grown overnight in M9 minimal medium supplemented with casamino acids, vitamin B1 and 0.4% sugar (glucose, sorbitol or lactose). The media was supplemented with kanamycin. Overnight cultures were diluted 1:100 in the respective fresh M9 medium and cultures were grown until specific growth phase. Snap shot were taken from the cultures at OD_600_ 0.2 to 0.25 for early log (EL), 0.5 to 0.55 for mid log (ML), 1 to 1.1 for stationary phase (SP) and after overnight growth (ON). To follow EI localization in overnight-grown cells that were inoculated into fresh medium, MG1655Φ(*ptsI*-mCherry) cells, which were grown overnight in M9 minimal media supplemented with CAA, vitamin B1, and glucose, were diluted and spotted onto 1% agar pads in the same medium, which had been pre-equilibrated to 30°C, and imaged immediately by time-lapse microscopy. To measure the correlation of 2-NBDG [(2-(N-(7-Nitrobenz-2-oxa-1,3-diazol-4-yl)Amino)-2-Deoxyglucose] uptake vs. EI-mCherry spatiotemporal organization, MG1655Φ(*ptsI*-mCherry) cells were grown overnight in M9 minimal medium supplemented with casamino acids, vitamin B1 and 0.4% glucose. Cells were pelleted, washed thrice and incubated for 10 min in M9 minimal medium supplemented with casamino acids, vitamin B1 and 10 μM 2-NBDG. After 10 min, cells were washed thrice and imaged in the microscope. To measure the correlation of *Pnag* mCerulean expression vs. EI-mCherry spatiotemporal organization, NTS102 cells were grown overnight in M63 minimal media supplemented with CCA, vitamin B1 and Glycerol (M63 glycerol) diluted 1:100 into M63 glycerol and grown till OD_600_ = 0.3–0.35. Cells were pelleted and resuspended in M63 supplemented with vitamin B1 and NAG. Snap shot in phase contrast, mCherry and mCerulean (CFP) channels were taken at time 0 and after 1 h.

### Fluorescence microscopy

Fluorescence microscopy was carried out as described previously (Govindarajan et al., 2013). For snap-shot imaging, 0.5 ml cells were centrifuged, washed with 1X phosphate buffered saline (PBS) and finally resuspended in 10–100 μl of PBS. Light microscopy was performed on a Nikon Eclipse Ti-E inverted microscope equipped with Perfect Focus System (PFS), CFI PLAN Apochromat DM 100X (numeric aperture 1.45) oil Ph3 objective and ORCA Flash 4 camera (Hamamatsu photonics). Chroma filter cubes were used as follows: ET-GFP for GFP and 2-NBDG, and ET-mCherry for mCherry. Time-lapse imaging was performed with the aid of OKOLAB cage incubator. Unless otherwise indicated, cells were spotted on 1% agar pads containing M9 media supplemented with glucose, sorbitol or lactose. Agar pads were pre-equilibrated to the appropriate temperature and cells were imaged by time-lapse microscopy at the respective temperature. For each experiment, all images were collected using uniform parameters of magnification and exposure. Images were processed using NIS Elements-AR (Nikon). Two-dimensional trajectories of dynamical clusters were obtained by processing the time-lapse images using Fiji in combination with its Manual Tracking plugin (Schindelin et al., 2012). Correlations were calculated using MATLAB. Statistical tests were performed using GraphPad Prism.

For FRAP microscopy, Nikon A1R confocal microscope equipped with Apochromat 60X objective (numeric aperture 1.4) was used. Photobleaching was done over the area in which the EI cluster is localized. Recovery was measured every 10 s for a total period of 10 min. Mean fluorescence intensity was normalized to the total fluorescence intensity for each ROI after bleaching. Images were analyzed using NIS Elements AR module.

#### Image analysis

Unless otherwise indicated, image analysis was done by NIS-Elements Advanced Research (AR) version 4.4 software (Nikon).

#### Analyzing of EI cluster size

Regions of interest (ROIs) were selected to mark the cluster region of the first image in each time-lapse series as following. First the details of the mCherry-acquired image were enhanced using the “Homogenization” tool. Next, binary layers were created using the “Define Threshold” tool. ROI were then drawn over the binary layers and copied onto the original mCherry image and the area of each ROI was exported. Finally, cells with marked ROI were manually sorted according to their EI dynamic pattern (non-Dyn, Dyn-1P or Dyn-OP). Scatter plot of the cluster area value for each pattern was obtained using GraphPad Prism. The statistical significance of the differences among cells with detectable clusters was determined by ordinary one-way ANOVA test using GraphPad Prism.

#### Calculating the fraction of each dynamic group

Time-lapse of individual cells were manually classified based on their EI dynamic pattern (non-Dyn, Dyn-1P, Dyn-OP or UC). The results of this analysis are presented as a pie chart or a stacked column chart. The statistical significance of the differences among cells with detectable clusters were determined by ordinary one-way ANOVA test using GraphPad Prism.

#### Measuring the correlation between cluster speed and cluster size

EI-mCherry clusters were marked throughout each time-lapse series by binary layer by using the “Define Threshold” tool. Clusters marked by binary layer were tracked using the “Track Binaries” tool of the NIS-Elements AR “Tracking” module. The binary center was tracked from the end to the beginning without any skip. For other features, we used the default parameters of the program. Values for average cluster speed and cluster area, which were obtained from the Tracking analysis, were plotted and displayed as a scatter plot. Spearman correlation was calculated using MATLAB corr function.

#### Measuring the correlation between cluster area and cell SDI

First, ROIs were selected to mark the cells outline as following. Binary layers were created using the “Define Threshold” tool over the phase contrast images. ROI were then drawn over the binary layers. Next, EI-mCherry clusters were marked by binary layer by using “Define Threshold” tool. Then the binary lyres were copied onto the original mCherry images. Finally, mCherry images data were exported. The SDI of the ROIs, which were drawn over the cell outline, were plotted and correlated with the MI of the binary layers, which were drawn over the cluster, using Microsoft Excel and MATLAB corr function.

#### Measuring cells arrea EI-mCerry concentration and distribution

ROIs were selected to mark the cells outline throughout time-lapse series of cells with undetectable clusters as following. ROI were selected on the first phase contrast image in each time-lapse series, using the “Define New ROI” tool of the “Tracking” module. Then the tool “Track Autodetected ROIs” of the “Tracking” module was used to outline the selected cells by appropriate ROI for each time point throughout the time-lapse series. The data were exported from the mCherry-acquired image and plotted using MATLAB custom made script.

#### Calculating the distribution of EI clusters from cell cycle-synchronized population

SUT201, which expresses EI-mCherry and ZapA-GFP from the chromosome, was used. Time-lapse images were acquired every 3 min for a total period of 3 h. From these images, newly born daughter cells, in which EI clusters were not detected, were manually monitored. The time at which a clear Z-ring was observed was noted and this was considered time 0 for the newly born daughter cells. The time-lapse series of individual cells were monitored and the time at which new clusters were formed was noted. Using the NIS Elements AR module, the cell length at the time of new cluster formation was measured.

#### Calculating the fraction of cells with detectable EI clusters

For each growth condition, 400 or more cells from three independent experiments were manually classified as having or not having a detectable EI-mCherry cluster. The statistical significance of the differences among cells with detectable clusters were determined by ordinary one-way ANOVA test using GraphPad Prism.

#### Following EI localization in overnight-grown cells inoculated into fresh

medium: ROIs were selected to mark the cells outline of the first image in each time-lapse series as following. Binary layers were created using the “Define Threshold” tool over the phase contrast acquired images. ROI were then drawn over the binary layers. From each ROI, the value of SDI from the mCherry channel, taken at time 0, for each cell were obtained and presented as a heat map. Each cell outlined by ROI was manually monitored and noted for each time point of the time-lapse series as having or not having a detectable EI-mCherry cluster until cell division was completed. At the final time point, the sister cells were classified depending on whether both had detectable clusters or not. Finally, the cells were aligned according to their EI-mCherry SDI values.

#### The relation between 2-NBDG uptake and EI-mCherry spatiotemporal organization

ROIs were selected to mark the cells outline of the first image in each time-lapse series as following. Binary layers were created using the “Define Threshold” tool over the phase contrast images. ROI were then drawn over the binary layers. From each ROI, the 2-NBDG MI value and the SDI values of the mCherry signal were exported and presented as a scatter plot.

### Western blotting

Equal amount of samples were collected, washed and their proteins were separated on 10% SDS–polyacrylamide gels. Gels were subjected to Western blot analysis as described previously (Lopian et al., 2010). α-GroEL (Abcam) and α-mCherry (Abcam) were used for detection of GroEL and EI-mCherry proteins, respectively.

## Author contributions

SG, NA, and OA-C contributed to the design of the study and wrote the manuscript; SG, NA, and TS contributed to data acquisition; SG, NA, TS, AN-S, and OA-C contributed to data interpretation.

### Conflict of interest statement

The authors declare that the research was conducted in the absence of any commercial or financial relationships that could be construed as a potential conflict of interest.

## References

[B1] Alley M. R. Maddock J. R. Shapiro L. (1992). Polar localization of a bacterial chemoreceptor. Genes Dev. 6, 825–836. 10.1101/gad.6.5.825 1577276

[B2] Amster-Choder O. (2011). The compartmentalized vessel: the bacterial cell as a model for subcellular organization (a tale of two studies). Cell. Logist. 1, 77–81. 10.4161/cl.1.2.16152 21686257 PMC3116588

[B3] Balaban N. Q. Merrin J. Chait R. Kowalik L. Leibler S. (2004). Bacterial persistence as a phenotypic switch. Science 305, 1622–1625. 10.1126/science.1099390 15308767

[B4] Barry R. M. Bitbol A.-F. Lorestani A. Charles E. J. Habrian C. H. Hansen J. M.. (2014). Large-scale filament formation inhibits the activity of CTP synthetase. Elife 3:e03638. 10.7554/eLife.03638 25030911 PMC4126345

[B5] Bowman G. R. Comolli L. R. Zhu J. Eckart M. Koenig M. Downing K. H.. (2008). A polymeric protein anchors the chromosomal origin/ParB complex at a bacterial cell pole. Cell 134, 945–955. 10.1016/j.cell.2008.07.015 18805088 PMC2745220

[B6] Bowman G. R. Lyuksyutova A. I. Shapiro L. (2011). Bacterial polarity. Curr. Opin. Cell Biol. 23, 71–77. 10.1016/j.ceb.2010.10.013 21095111 PMC7500059

[B7] Briegel A. Ortega D. R. Tocheva E. I. Wuichet K. Li Z. Chen S.. (2009). Universal architecture of bacterial chemoreceptor arrays. Proc. Natl. Acad. Sci. U.S.A. 106, 17181–17186. 10.1073/pnas.0905181106 19805102 PMC2761316

[B8] Deutscher J. Aké F. M. D. Derkaoui M. Zébré A. C. Cao T. N. Bouraoui H.. (2014). The bacterial phosphoenolpyruvate: carbohydrate phosphotransferase system: regulation by protein phosphorylation and phosphorylation-dependent protein-protein interactions. Microbiol. Mol. Biol. Rev. 78, 231–256. 10.1128/MMBR.00001-14 24847021 PMC4054256

[B9] Deutscher J. Francke C. Postma P. W. (2006). How phosphotransferase system-related protein phosphorylation regulates carbohydrate metabolism in bacteria. Microbiol. Mol. Biol. Rev. 70, 939–1031. 10.1128/MMBR.00024-06 17158705 PMC1698508

[B10] Ebersbach G. Briegel A. Jensen G. J. Jacobs-Wagner C. (2008). A self-associating protein critical for chromosome attachment, division, and polar organization in caulobacter. Cell 134, 956–968. 10.1016/j.cell.2008.07.016 18805089 PMC2614312

[B11] Elowitz M. B. Levine A. J. Siggia E. D. Swain P. S. (2002). Stochastic gene expression in a single cell. Science 297, 1183–1186. 10.1126/science.1070919 12183631

[B12] Franklin T. J. Godfrey A. (1965). Resistance of *Escherichia coli* to tetracyclines. Biochem. J. 94:54. 14345884 10.1042/bj0940054 PMC1206404

[B13] Galli E. Gerdes K. (2010). Spatial resolution of two bacterial cell division proteins: ZapA recruits ZapB to the inner face of the Z-ring. Mol. Microbiol. 76, 1514–1526. 10.1111/j.1365-2958.2010.07183.x 20487275

[B14] Gefen O. Balaban N. Q. (2009). The importance of being persistent: heterogeneity of bacterial populations under antibiotic stress. FEMS Microbiol. Rev. 33, 704–717. 10.1111/j.1574-6976.2008.00156.x 19207742

[B15] Govindarajan S. Elisha Y. Nevo-Dinur K. Amster-Choder O. (2013). The general phosphotransferase system proteins localize to sites of strong negative curvature in bacterial cells. MBio 4:e00443–13. 10.1128/mBio.00443-13 24129255 PMC3812706

[B16] Govindarajan S. Nevo-Dinur K. Amster-Choder O. (2012). Compartmentalization and spatiotemporal organization of macromolecules in bacteria. FEMS Microbiol. Rev. 36, 1005–1022. 10.1111/j.1574-6976.2012.00348.x 22775310

[B17] Holland S. L. Reader T. Dyer P. S. Avery S. V. (2014). Phenotypic heterogeneity is a selected trait in natural yeast populations subject to environmental stress. Environ. Microbiol. 16, 1729–1740. 10.1111/1462-2920.12243 24000788 PMC4231229

[B18] Huitema E. Pritchard S. Matteson D. Radhakrishnan S. K. Viollier P. H. (2006). Bacterial birth scar proteins mark future flagellum assembly site. Cell 124, 1025–1037. 10.1016/j.cell.2006.01.019 16530048

[B19] Ingerson-Mahar M. Briegel A. Werner J. N. Jensen G. J. Gitai Z. (2010). The metabolic enzyme CTP synthase forms cytoskeletal filaments. Nat. Cell Biol. 12, 739–746. 10.1038/ncb2087 20639870 PMC3210567

[B20] Izoré T. van den Ent F. (2017). Bacterial Actins. Subcell. Biochem. 84, 245–266. 10.1007/978-3-319-53047-5_8 28500528

[B21] Janakiraman A. Goldberg M. B. (2004). Recent advances on the development of bacterial poles. Trends Microbiol. 12, 518–525. 10.1016/j.tim.2004.09.003 15488393

[B22] Jensen R. B. Wang S. C. Shapiro L. (2002). Dynamic localization of proteins and DNA during a bacterial cell cycle. Nat. Rev. Mol. Cell Biol. 3, 167–176. 10.1038/nrm758 11994737

[B23] Kotte O. Volkmer B. Radzikowski J. L. Heinemann M. (2014). Phenotypic bistability in *Escherichia coli* 9s central carbon metabolism. Mol. Syst. Biol. 10:736 10.15252/msb.20135022 24987115 PMC4299493

[B24] Laloux G. Jacobs-Wagner C. (2014). How do bacteria localize proteins to the cell pole? J. Cell Sci. 127, 11–19. 24345373 10.1242/jcs.138628 PMC3874780

[B25] Lam H. Schofield W. B. Jacobs-Wagner C. (2006). A landmark protein essential for establishing and perpetuating the polarity of a bacterial cell. Cell 124, 1011–1023. 10.1016/j.cell.2005.12.040 16530047

[B26] Lenarcic R. Halbedel S. Shaw M. Wu L. J. Errington J. Marenduzzo D.. (2009). Localisation of DivIVA by targeting to negatively curved membranes. EMBO J. 28, 2272–2282. 10.1038/emboj.2009.129 19478798 PMC2690451

[B27] Lopian L. Elisha Y. Nussbaum-Shochat A. Amster-Choder O. (2010). Spatial and temporal organization of the *E. coli* PTS components. EMBO J. 9, 3630–3645. 10.1038/emboj.2010.240 PMC2982763 20924357

[B28] Losick R. Desplan C. (2008). Stochasticity and cell fate. Science 320, 65–68. 10.1126/science.1147888 18388284 PMC2605794

[B29] Lutkenhaus J. (2007). Assembly dynamics of the bacterial MinCDE system and spatial regulation of the Z ring. Annu. Rev. Biochem. 76, 539–562. 10.1146/annurev.biochem.75.103004.142652 17328675

[B30] Lüttmann D. Göpel Y. Görke B. (2015). Cross-talk between the canonical and the nitrogen-related phosphotransferase systems modulates synthesis of the KdpFABC potassium transporter in *Escherichia coli*. J. Mol. Microbiol. Biotechnol. 25, 168–177. 10.1159/000375497 26159077

[B31] Michaelis A. M. Gitai Z. (2010). Dynamic polar sequestration of excess MurG may regulate enzymatic function. J. Bacteriol. 192, 4597–4605. 10.1128/JB.00676-10 20644141 PMC2937405

[B32] Oliva M. A. Halbedel S. Freund S. M. Dutow P. Leonard T. A. Veprintsev D. B.. (2010). Features critical for membrane binding revealed by DivIVA crystal structure. EMBO J. 29, 1988–2001. 10.1038/emboj.2010.99 20502438 PMC2892376

[B33] Parry B. R. Surovtsev I. V. Cabeen M. T. O'Hern C. S. Dufresne E. R. Jacobs-Wagner C. (2014). The bacterial cytoplasm has glass-like properties and is fluidized by metabolic activity. Cell 156, 183–194. 10.1016/j.cell.2013.11.028 24361104 PMC3956598

[B34] Peng B. Su Y.-B. Li H. Han Y. Guo C. Tian Y.-M.. (2015). Exogenous alanine and/or glucose plus kanamycin kills antibiotic-resistant bacteria. Cell Metab. 21, 249–261. 10.1016/j.cmet.2015.01.008 25651179

[B35] Peters N. T. Dinh T. Bernhardt T. G. (2011). A fail-safe mechanism in the septal ring assembly pathway generated by the sequential recruitment of cell separation amidases and their activators. J. Bacteriol. 193, 4973–4983. 10.1128/JB.00316-11 21764913 PMC3165665

[B36] Ramamurthi K. S. Losick R. (2009). Negative membrane curvature as a cue for subcellular localization of a bacterial protein. Proc. Natl. Acad. Sci. U.S.A. 106, 13541–13545. 10.1073/pnas.0906851106 19666580 PMC2726380

[B37] Rudner D. Z. Losick R. (2010). Protein subcellular localization in bacteria. Cold Spring Harb. Perspect. Biol. 2:a000307. 10.1101/cshperspect.a000307 20452938 PMC2845201

[B38] Schindelin J. Arganda-Carreras I. Frise E. Kaynig V. Longair M. Pietzsch T.. (2012). Fiji: an open-source platform for biological-image analysis. Nat. Methods 9, 676–682. 10.1038/nmeth.2019 22743772 PMC3855844

[B39] Strahl H. Ronneau S. González B. S. Klutsch D. Schaffner-Barbero C. Hamoen L. W. (2015). Transmembrane protein sorting driven by membrane curvature. Nat. Commun. 6:8728. 10.1038/ncomms9728 26522943 PMC4632190

[B40] Studdert C. A. Parkinson J. S. (2005). Insights into the organization and dynamics of bacterial chemoreceptor clusters through *in vivo* crosslinking studies. Proc. Natl. Acad. Sci. U.S.A. 102, 15623–15628. 10.1073/pnas.0506040102 16230637 PMC1266109

[B41] Thiem S. Sourjik V. (2008). Stochastic assembly of chemoreceptor clusters in *Escherichia coli*. Mol. Microbiol. 68, 1228–1236. 10.1111/j.1365-2958.2008.06227.x 18476921

[B42] Thiem S. Kentner D. Sourjik V. (2007). Positioning of chemosensory clusters in *E. coli* and its relation to cell division. EMBO J. 26, 1615–1623. 10.1038/sj.emboj.7601610 17332753 PMC1829377

[B43] Treuner-Lange A. Søgaard-Andersen L. (2014). Regulation of cell polarity in bacteria. J. Cell Biol. 206, 7–17. 10.1083/jcb.201403136 25002676 PMC4085708

[B44] Wang X. Kang Y. Luo C. Zhao T. Liu L. Jiang X.. (2014). Heteroresistance at the single-cell level: adapting to antibiotic stress through a population-based strategy and growth-controlled interphenotypic coordination. MBio 5:e00942–13. 10.1128/mBio.00942-13 24520060 PMC3950525

[B45] Westermayer S. A. Fritz G. Gutiérrez J. Megerle J. A. Weißl M. P. S. Schnetz K.. (2016). Single-cell characterization of metabolic switching in the sugar phosphotransferase system of *Escherichia coli*. Mol. Microbiol. 100, 472–485. 10.1111/mmi.13329 26784570

[B46] Yamada M. Saier M. H. (1988). Positive and negative regulators for glucitol (gut) operon expression in *Escherichia coli*. J. Mol. Biol. 203, 569–583. 10.1016/0022-2836(88)90193-3 3062173

[B47] Yoshioka K. Saito M. Oh K. B. Nemoto Y. Matsuoka H. Natsume M.. (1996). Intracellular fate of 2-NBDG, a fluorescent probe for glucose uptake activity, in *Escherichia coli* cells. Biosci. Biotechnol. Biochem. 60, 1899–1901. 10.1271/bbb.60.1899 8987871

[B48] Yu J. Liu Y. Chang Z. (2017). An organelle-like structure correlated with the quiescent state of bacterial cells. bioRxiv. 10.1101/107466

