# Supplementary material for: Phenotypic Heterogeneity in Sugar Utilization by E. coli Is Generated by Stochastic Dispersal of the General PTS Protein EI from Polar Clusters
Source: Front Microbiol. 2018 Jan 17;8:2695. doi: 10.3389/fmicb.2017.02695 (PMC5776091; doi:10.3389/fmicb.2017.02695)
Supplement: Supplementary file 2 [file DataSheet1.pdf]

SUPPLEMENTAL INFORMATION

Phenotypic heterogeneity in sugar utilization by *E. coli* is generated by stochastic dispersal of the general PTS protein EI from polar clusters

Sutharsan Govindarajan*, Nitsan Albocher*, Tamar Szoke, Anat Nussbaum-Shohat and Orna Amster-Choder

Supplemental Figure 1

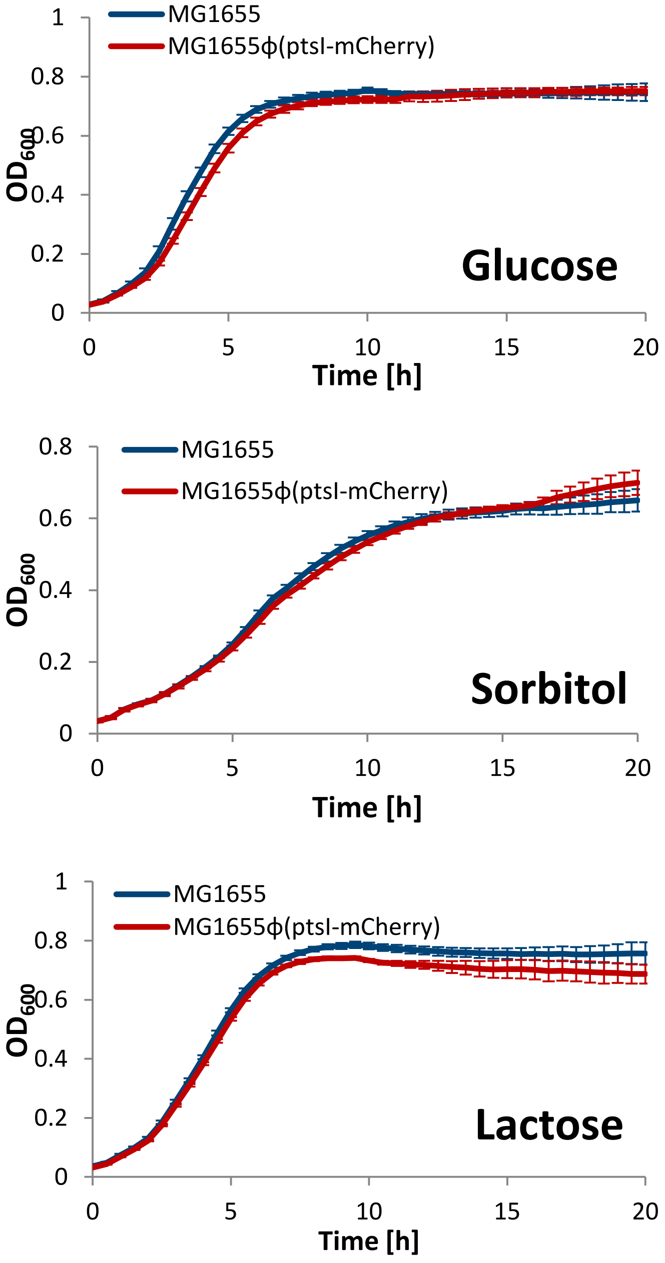

Fig. S1. The growth rate of strains expressing EI or EI-mCherry from their chromosome is comparable in media supplemented with PTS or non-PTS sugars.

Shown are growth curves of the wild-type strain (MG1655) and of a strain expressing an EI-mCherry chromosomal fusion MG1655Φ(*ptsI-*mCherry*),* in M9 medium supplemented with PTS sugars (glucose or sorbitol) or with a non PTS sugar (lactose).

Supplemental Figure 2

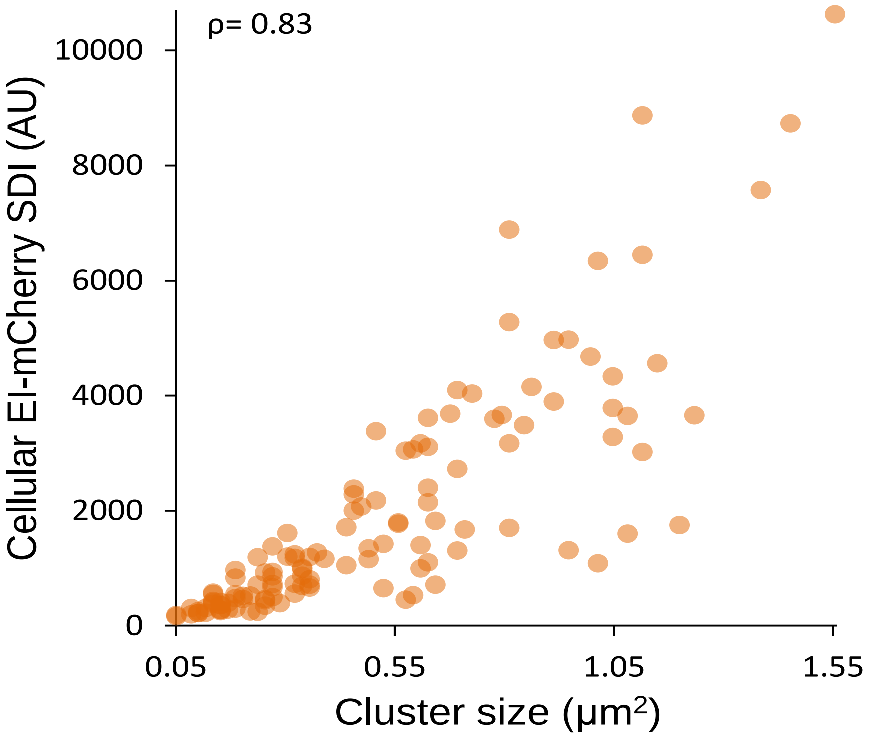

Fig. S2. Direct correlation between EI-mCherry cellular distribution and cluster size.

Scatter plot of cellular EI-mCherry standard division intensity (SDI) (AU) versus cluster area (μm^2), referred to as cluster size (n=124). Pearson correlation ρ=0.83; *p*-value= 7.89E-34.

Supplemental Figure 3

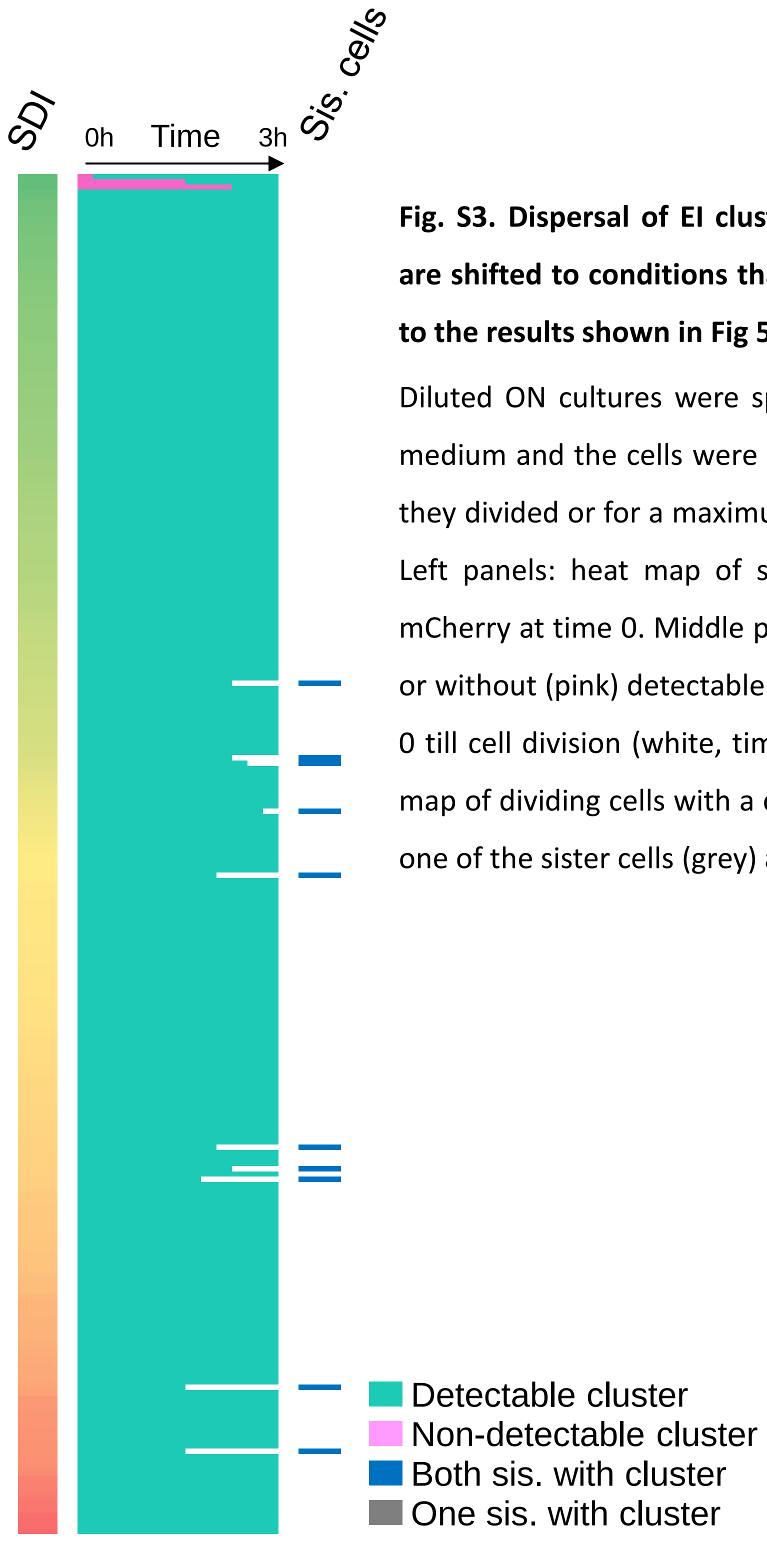

Fig. S3. Dispersal of EI clusters in ON cells occurs only when cells are shifted to conditions that enable growth (a control experiment to the results shown in Fig 5A).

Diluted ON cultures were spotted on an agar pad with filtered ON medium and the cells were observed at time 0 and every 15 min till they divided or for a maximum of 3 hours, by time-lapse microscopy. Left panels: heat map of standard deviation intensity (SDI) of EI-mCherry at time 0. Middle panels: heat map of cells with (turquoise) or without (pink) detectable EI-mCherry clusters over time from time 0 till cell division (white, time after cell division). Right panels: Heat map of dividing cells with a cluster in each sister cell (blue) or only in one of the sister cells (grey) after 3 hours.

Supplemental Figure 4

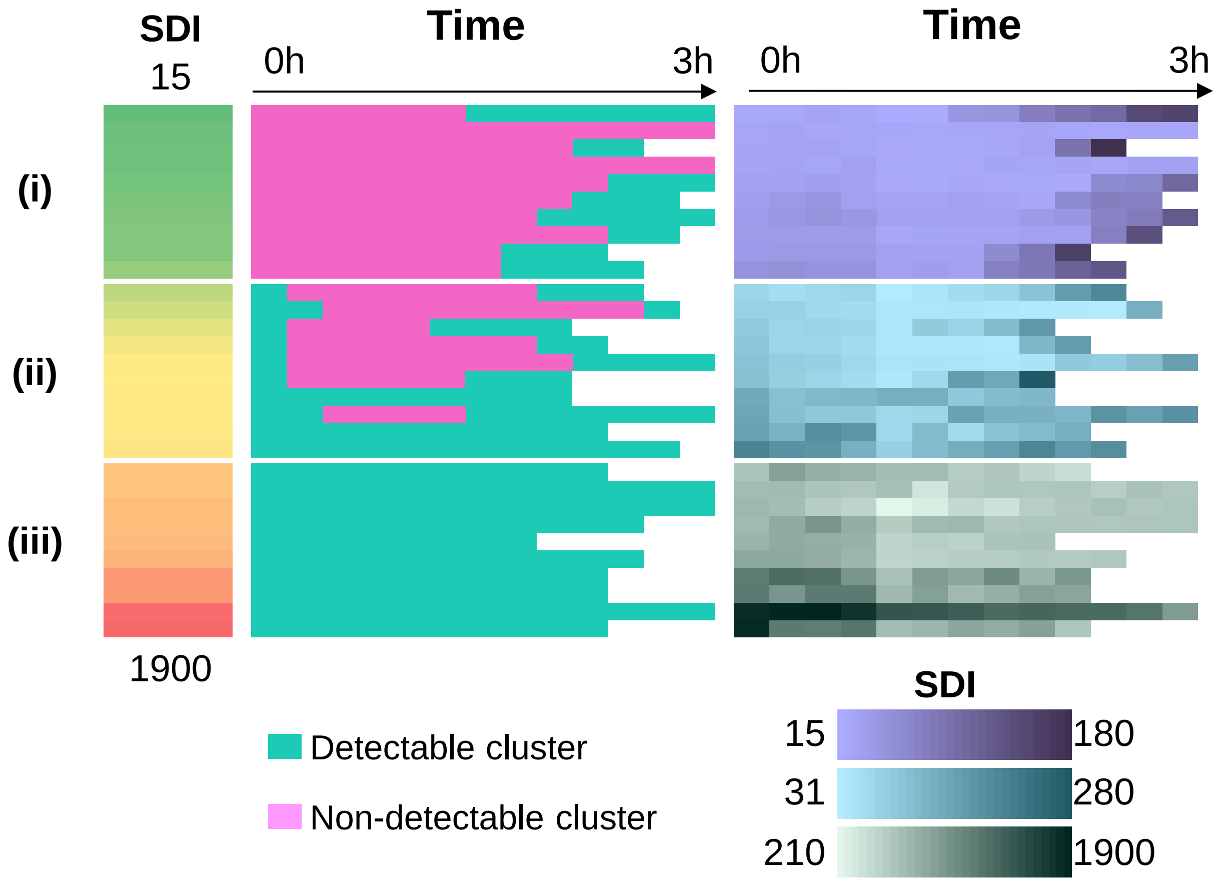

Fig. S4. Dispersal of EI clusters after the transition of ON cells to fresh medium is a gradual process.

Diluted ON cultures were spotted on an agar pad with fresh medium and the cells were observed at time 0 and every 15 min till they divided or for a maximum of 3 hours, by time-lapse microscopy. Left panels: heat map of standard deviation intensity (SDI) of EI-mCherry at time 0. Middle panels: heat map of cells with (turquoise) or without (pink) detectable EI-mCherry clusters over time, from time 0 till cell division (white, time after cell division). Right panels: Heat map of standard deviation intensity (SDI) of EI-mCherry over time, from time 0 till cell division (white, time after cell division). The data is divided to 3 sub-populations, as suggested by the results in Fig. 5A : i) cells with no detectable clusters at time 0 (purple shade). ii) cells with relatively small clusters at time 0 that dispersed during growth and or did not disperse till they divided. iii) cells with big clusters at time 0.

Supplemental Figure 5

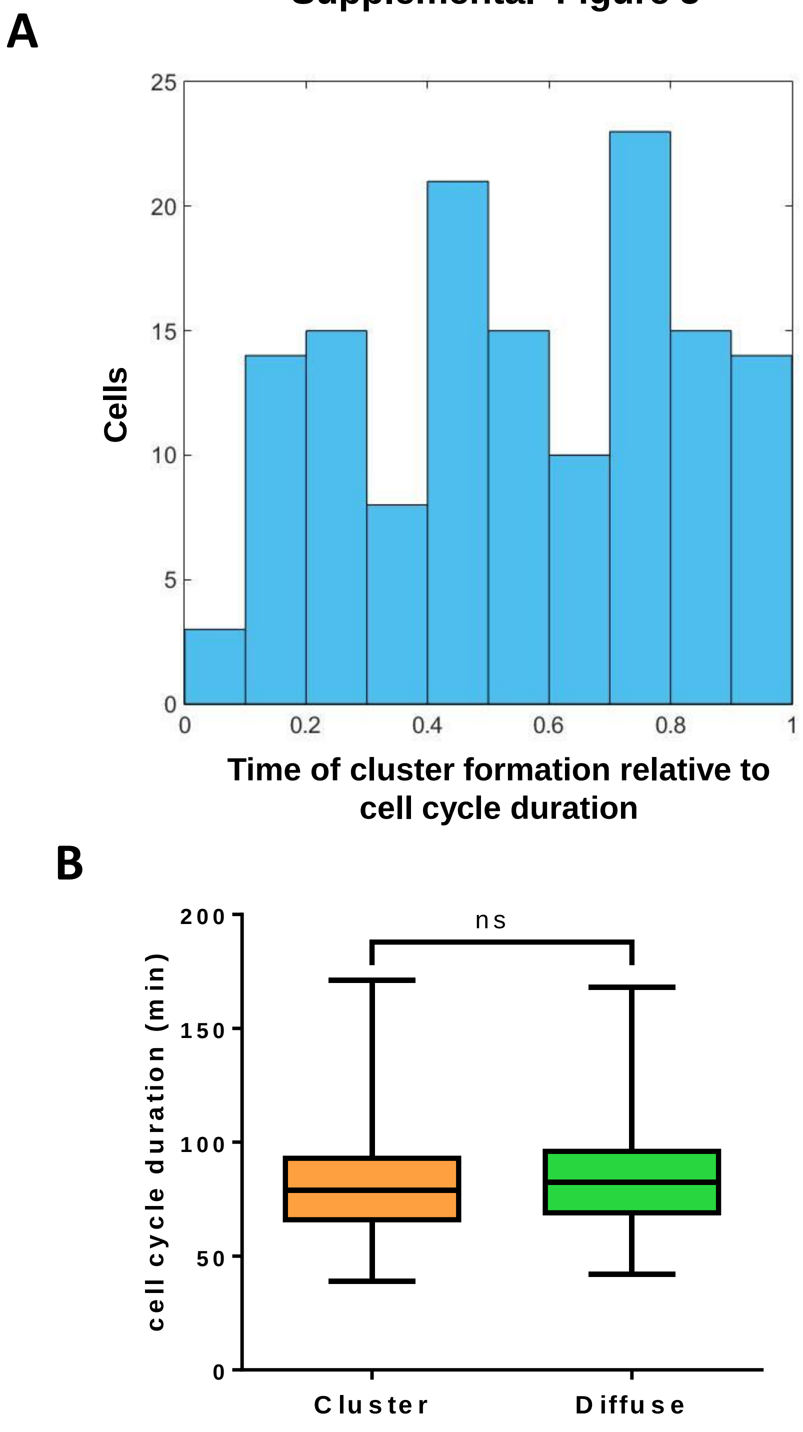

Fig. S5. Cluster formation is not linked to cell cycle.

(A) A histogram showing the distribution of the time it takes a new cluster to form relative to cell cycle duration (n=138).

(B) A box plot showing cell cycle duration of cells with EI cluster (orange box; n=138) or with diffuse EI (green box; n=138) at the beginning of the cell cycle. Unpaired t-test; *p*-value= 0.45
